# Unlocking the Potential of *Rosmarinus officinalis* L.: Modern and Sustainable Strategies for the Extraction of Bioactive Metabolites for Cosmetic and Functional Food Applications

**DOI:** 10.3390/foods15183247

**Published:** 2026-09-14

**Authors:** Abraham Josué Nevárez-Ramírez, Mónica I. García-Aranda, Rene Cabezas, Roberto Castro-Muñoz

**Affiliations:** 1 Laboratorio de Inmunofarmacología, Unidad Profesional Interdisciplinaria de Biotecnología del Instituto Politécnico Nacional, Av. Acueducto s/n Barrio la Laguna Ticomán, Ciudad de México 07340, Mexico; anevarez@ipn.mx (A.J.N.-R.);; 2 Departamento de Química Ambiental, Facultad de Ciencias, Universidad Católica de la Santísima Concepción, Concepción 4090541, Chile; rene.cabezas@ucsc.cl; 3 Institute of Sustainable Processes, University of Valladolid, Dr. Mergelina s/n., 47011 Valladolid, Spain; 4 Department of Sanitary Engineering, Faculty of Civil and Environmental Engineering, Gdansk University of Technology, G. Narutowicza St. 11/12, 80-233 Gdansk, Poland

**Keywords:** rosemary, bioactive metabolites, phenolic compounds, essential oils, extraction technologies, functional foods, cosmetics

## Abstract

The food and cosmetics industries are showing increasing scientific interest in plant-based bioactive compounds, positioning rosemary (*Rosmarinus officinalis* L.) as a high-value source of phytochemicals with antioxidant, antimicrobial, anti-inflammatory, and neuroprotective properties. This review analyzes the principal methods used to extract rosemary bioactive metabolites, comparing conventional techniques with emerging approaches in terms of extraction efficiency, sustainability, and preservation of bioactive compounds. The discussion covers hydrodistillation, steam distillation, Soxhlet extraction, and supercritical fluid extraction, as well as emerging technologies such as ultrasound, microwave, pulsed electric field, enzyme-assisted extraction, and deep eutectic solvents. Particular attention is given to how extraction strategies influence the recovery and stability of major compounds, including carnosic acid, carnosol, rosmarinic acid, 1,8-cineole, and α-pinene, considering their different physicochemical properties. Overall, emerging technologies may improve extraction yield or reduce processing time under specific conditions; however, their environmental performance and ability to preserve compound stability depend on solvent selection, energy and water consumption, process configuration, and downstream requirements. These advances support the sustainable production of rosemary-derived ingredients with promising applications in functional foods and cosmetic formulations.

## 1. Introduction

The scientific and technological interest in the use of plant-derived bioactive compounds has grown significantly in recent years, driven by demand for natural alternatives in the food and cosmetic industries. In this context, rosemary, currently accepted as *Salvia rosmarinus* Spenn. (syn. *Rosmarinus officinalis* L.; hereafter referred to as *R. officinalis*), a perennial aromatic plant in the Lamiaceae family, native to the Mediterranean region and widely cultivated, has gained relevance due its rich phytochemical composition. This species, which can reach approximately one meter in height and has white or bluish flowers, stands out for its biological properties attributed to the presence of terpenes, phenolic acids, flavonoids, and essential oils [1,2].

**Figure 1:**
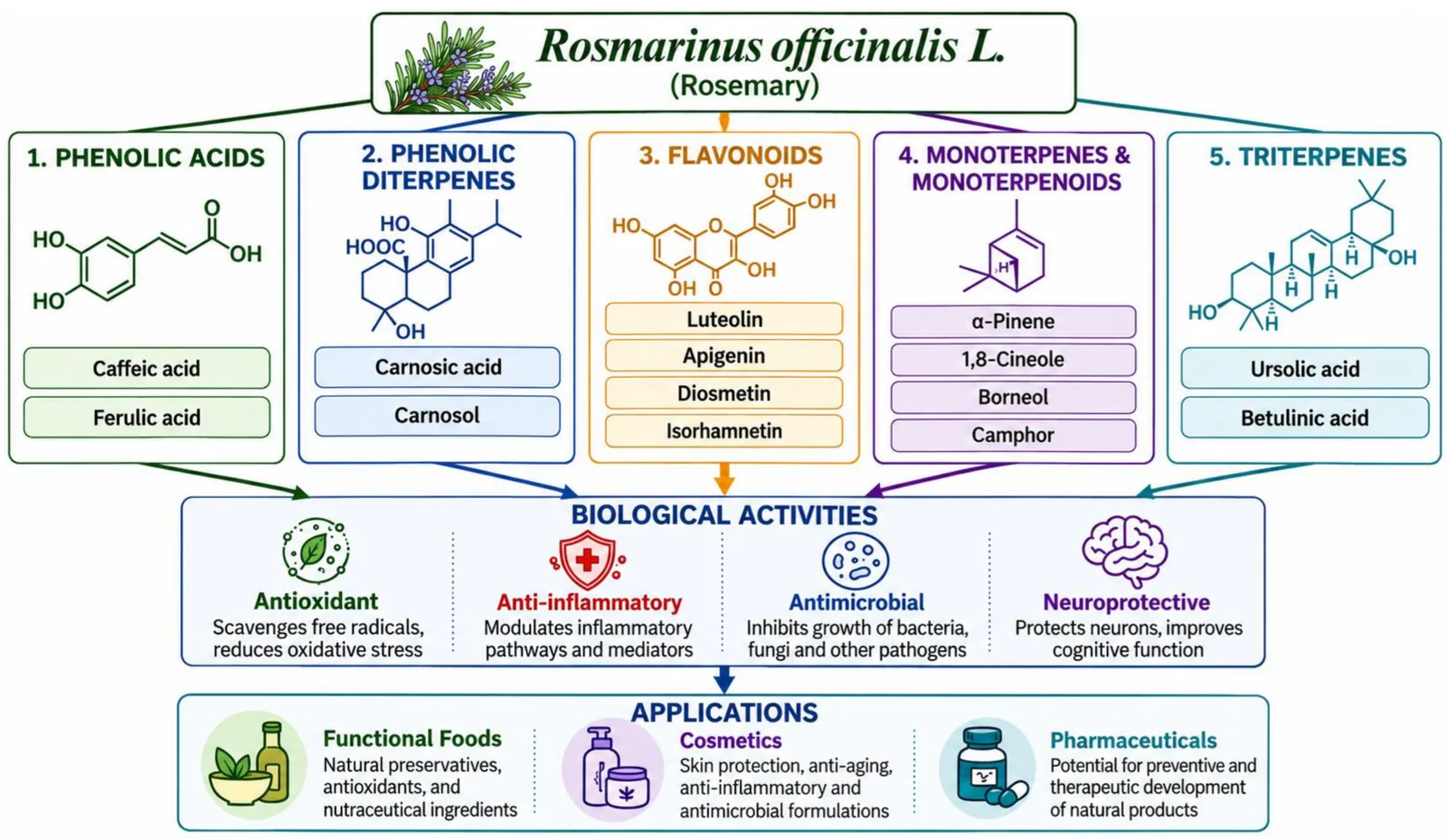
Main groups of secondary metabolites present in *Rosmarinus officinalis* L. and their relationship with biological activities and industrial applications.

Several studies have indicated that rosemary has a high concentration of secondary metabolites. Its phytochemical composition includes phenolic compounds, non-volatile terpenoids, and a volatile fraction rich in monoterpenes and monoterpenoids, which together explain its broad spectrum of biological activities (Figure 1). Among the main metabolites identified are phenolic compounds (rosmarinic acid and caffeic acid) and flavonoids (cirsimaritin and diosmetin) [3], non-volatile terpenoids, including carnosic acid, carnosol, ursolic acid, betulinic acid, and their derivatives; as well as the components of essential oils, represented by monoterpenes (α-pinene, camphene, and limonene) and monoterpenoids (1,8-cineole, borneol, verbenone, and camphor).

Although rosemary (*Rosmarinus officinalis* L.) is widely known for its high content of phenolic compounds such as rosmarinic acid and flavonoids like luteolin and apigenin, it also contains secondary metabolites present in much lower concentrations that could play a significant role in its biological properties. Among these minor compounds are isorhamnetin [3], identified as the only flavonol present in certain extracts, as well as lignans such as medioresinol and its derivatives [4]. These less abundant metabolites, although frequently overlooked, could contribute synergistically to the therapeutic effect of rosemary and represent a promising area for future phytochemical and pharmacological research.

Beyond these phytochemicals, rosemary processing by-products also constitute an important source of bioactive compounds. The aqueous and solid residues contain valuable compounds such as rosmarinic acid and carnosol, exhibiting promising biological properties [5]. Rosemary extracts and essential oils have shown activity against both Gram-positive and Gram-negative bacteria, such as *Staphylococcus aureus*, *Escherichia coli*, *Pseudomonas aeruginosa*, and *Klebsiella pneumoniae*, attributed to disrupting cell membranes and inhibiting enzymatic activity [6]. Its antifungal properties against *Candida albicans*, *Aspergillus niger*, and *Penicillium chrysogenum* have been associated with disruption of fungal membranes and interference with ergosterol biosynthesis, which is essential for maintaining membrane integrity. Rosemary has demonstrated antiviral potential against various viruses by interacting with the viral envelope, inhibiting viral replication, and protecting against infection [7,8]. The chemical composition and biological activities of plant extracts depend on numerous abiotic factors, such as soil, climate, and season.

To harness the bioactive potential of rosemary, it is essential to select appropriate extraction methods that allow for obtaining the compounds of interest with the greatest possible efficiency, stability, and purity. While traditional techniques, such as hydrodistillation yielding a composition rich in 1,8-cineole, α-pinene, and camphor [9], and steam distillation, have been widely used for obtaining essential oils, emerging techniques such as ultrasound-assisted extraction, microwave extraction, and pulsed electric field extraction are gaining prominence because they may intensify mass transfer and reduce processing time under appropriate conditions; however, their extraction performance and environmental advantages remain process- and scale-dependent [10,11].

This review aims to analyze and compare the main, advanced and emerging methods for extracting bioactive metabolites from rosemary, with an emphasis on their applicability in the development of functional foods and cosmetic products. The advantages and limitations of each technique are discussed, as well as their efficiency in recovering specific compounds based on their polarity and thermal stability. It is worth mentioning that purification and fractionation approaches are considered when they constitute complementary downstream operations directly associated with the recovery or selective enrichment of the target metabolites. Overall, this review seeks to provide a useful guide for researchers and industries interested in valorizing this aromatic plant as a natural source of functional ingredients.

## 2. Literature Search Strategy

This narrative review was based on searches conducted in Scopus, Web of Science, PubMed, ScienceDirect, and Google Scholar. The search focused on peer-reviewed articles and reviews published in English between January 2020 and July 2026. Keywords included “*Rosmarinus officinalis*”, “*Salvia rosmarinus*”, “rosemary”, “bioactive compounds”, “phenolic compounds”, “terpenes”, “essential oil”, “extraction”, “purification”, “fractionation”, “green extraction”, “functional foods”, and “cosmetic applications”. Eligible publications addressed rosemary composition, extraction or purification technologies, and applications of rosemary-derived compounds. Theses, book chapters, conference abstracts, non-peer-reviewed documents, and publications in languages other than English were excluded. Selected studies published before 2020 were retained when they provided foundational evidence on the physicochemical or biological properties of rosemary metabolites or compound concentrations relevant to comparison with recent findings. Publications were ultimately selected based on their relevance to the review scope and the completeness of the reported methodological and analytical information, enabling the available evidence to be interpreted within its experimental context.

## 3. Chemical Profiling of Rosemary Metabolites and Their Interest in Functional Foods and Cosmetics

Among rosemary phytochemicals, phenolic constituents have attracted particular attention due to their antioxidant, anti-inflammatory, antimicrobial, and preservative properties. According to the literature summarized by Kiss et al. [12], rosmarinic acid and the phenolic diterpene carnosic acid have been reported in rosemary at concentrations of up to 12.5 and 8.2 mg/g, respectively. However, the reporting basis for these concentrations—such as dry plant material, fresh material, or extract—was not specified in the cited source. Caffeic and ferulic acids have also been identified among the phenolic constituents of rosemary [12]. Rosmarinic acid, in particular, exhibits strong antioxidant activity and is widely used as a natural preservative in functional foods and anti-aging cosmetic formulations [13].

In food systems, rosemary extracts and their phenolic constituents have been investigated as natural antioxidants capable of delaying lipid oxidation and extending product shelf life [14,15]. In cosmetic research, their free-radical-scavenging activity has also been associated with potential protection against photoaging and UV-induced oxidative damage [16]. The volatile fraction of rosemary essential oil contains hydrocarbon monoterpenes, such as α-pinene, as well as oxygenated monoterpenes, including 1,8-cineole and camphor. Rosemary essential oil and selected volatile constituents have demonstrated antimicrobial and aromatic properties, supporting their potential use as flavoring or preservative agents in foods and as functional ingredients in cosmetic products [17,18,19,20,21]. Rosemary also contains flavonoids such as luteolin and apigenin, which contribute to its antioxidant and anti-inflammatory profile [22,23].

Taken together, these chemically diverse metabolites contribute to the biological and technological potential of rosemary. However, evidence obtained using isolated compounds, essential oils, and complete rosemary extracts should be interpreted separately, as their composition, concentration, and biological responses are not necessarily equivalent. Table 1 summarizes the principal secondary metabolites reported in *Rosmarinus officinalis L.*, their chemical classification, biological activities, and reported or potential applications in functional foods and cosmetic products. The concentration of each metabolite may vary considerably according to geographic origin, chemotype, harvest season, plant part, and extraction method.

The biological activities summarized in this section are supported by different levels of evidence, including studies on isolated compounds, rosemary extracts or essential oils, and rosemary-containing formulations. Activities demonstrated for an isolated metabolite indicate its intrinsic biological potential but cannot be directly extrapolated to a complete rosemary extract, as compound concentration, matrix interactions, bioavailability, and formulation characteristics may influence the final response. Therefore, potential applications are identified as such unless they have been experimentally demonstrated using a rosemary-derived extract or formulation.

## 4. Comparison of Extraction Methods Applied to Rosemary Metabolites: Efficiency, Selectivity and Sustainability

### 4.1. Factors Influencing the Recovery of Rosemary Metabolites

The extraction of bioactive metabolites from *Rosmarinus officinalis* L. is a key aspect of the phytochemical valorization of this species, due to the wide diversity of compounds present in its plant tissues. Among the most relevant metabolites, as discussed in the previous section, are monoterpenes, phenolic diterpenes, and phenolic acids, which exhibit distinct physicochemical properties that directly influence the efficiency of extraction processes. In this context, the extraction approaches reported for *Rosmarinus officinalis* L. were organized according to the principal type of fraction recovered. Table 2 summarizes distillation and other volatile-oriented approaches used for the recovery of essential oils and terpene constituents, whereas Table 3 presents solvent-based, assisted, and emerging technologies applied primarily to phenolic compounds and their fractionation. Together, Table 2 and Table 3 provide study-level summaries of the operating conditions, target compounds, principal outcomes, and critical considerations reported in individual studies. Purification or fractionation operations are included when they are directly integrated with extraction or contribute to the selective enrichment of the recovered metabolites. By contrast, the subsequent subsections discuss the principles, advantages, limitations, and potential applicability of each technology. The numerical values compiled in Table 2 and Table 3 are therefore presented descriptively and should not be interpreted as a direct ranking of extraction technologies.

In addition to the structural diversity of metabolites present in *Rosmarinus officinalis* L., the intracellular distribution of these compounds also significantly influences their recovery during extraction processes. Monoterpenes and other volatile compounds are usually found accumulated in glandular trichomes located primarily on the leaf surface, while phenolic compounds can be found in cell vacuoles or associated with cell wall structures [53,54]. This difference in anatomical location means that some metabolites can be released relatively easily through distillation processes, while others require conditions that favour the disruption of cell structures or solubilization with suitable solvents.

Furthermore, the rosemary plant matrix contains structural polysaccharides, lignin, and proteins that can act as physical barriers to the release of bioactive compounds [55]. For this reason, the efficiency of extraction processes depends not only on the chemical affinity between the metabolite and the solvent, but also on the ability of the method used to facilitate mass transfer between the plant matrix and the extraction medium. This aspect is particularly relevant when comparing conventional techniques with energy-assisted technologies, which can modify the cellular structure of the plant material and promote the diffusion of metabolites.

**Table 2:** Distillation and other volatile-oriented approaches reported for the recovery and purification of rosemary essential oil and terpene constituents.

Approach	Reported Recovery/Profile	Key Conditions	Critical Considerations	Ref.
**Clevenger hydrodistillation (CHD)**	Terpenes—Essential oil yield: 1.8%; 1,8-cineole: 60.96% after 20 min; α-pinene: 13.33% after 180 min.	20 g plant material; 300 mL distilled water; hydrodistillation, 120 min.	Simple and inexpensive, but prolonged heating may increase energy demand and alter heat-sensitive constituents.	[56]
	Terpenes—Maximum essential oil yield: 2.5%; 1,8-cineole: 25.26 ± 7.71%; camphor: 29.46 ± 4.92%; α-pinene: 12.34 ± 1.83%.	100 g dried leaves; 1500 mL distilled water; 250 °C; 180 min.	High reported yield, although the elevated processing temperature may increase energy demand and affect heat-sensitive constituents.	[57]
**Hydrodistillation followed by ethanolic extraction**	Essential oil yield: 1.57%.	70 g fresh leaves; 350 mL distilled water; 100 °C, 180 min; subsequent extraction with 80% ethanol at 70 °C for 60 min.	Sequential recovery can valorize both volatile and non-volatile fractions, but adds solvent, heat, and downstream separation requirements.	[58]
**Hydrodistillation (HD)**	Terpenes—Essential oil yield: 0.87%; 1,8-cineole: 51.79% in the rainy season; α-pinene: 12.8% in summer.	Aerial parts (leaves, stems, and flowers); hydrodistillation, 4 h.	Seasonal variation strongly affects composition; long extraction time increases thermal and energy burdens.	[59]
	Terpenes—Essential oil yield: 1.8%; 1,8-cineole: 52.44%; camphor: 11.24%; α-pinene: 8.23%.	100 g plant material; 1000 mL distilled water; 180 min.	Suitable for essential oils, but water and energy consumption should be considered.	[60]
**Steam distillation (SD)**	Terpenes—Essential oil yield: 6 mL per 1.25 kg plant material.	1.25 kg fresh material; 2.5 kg water containing (NH_4_)_2_SO_4_, (NH_4_)_3_PO_4_, and NaOH; steam distillation.	Reduced direct water contact, but salts and alkali complicate effluent handling and downstream purification.	[61]
	Terpenes—1,8-cineole: 34.25%; α-pinene: 20.98%; camphor: 13.75%.	100 g dried leaves; 900 mL distilled water; 180 min.	Appropriate for volatile recovery, although prolonged heating can cause losses or compositional changes.	[62]
	Terpenes—Essential oil yield: 2.31%; 1,8-cineole: 12.58%; camphor: 10.97%.	310 g aerial parts; 1240 mL distilled water; 180 min.	Performance depends on plant part and composition; direct comparison requires equivalent feed material.	[63]
	Terpenes—D-α-pinene: 22.1%; 1,8-cineole: 17.0%; verbenone: 14.9%.	Plant-to-water ratio 1:4 (*w*/*v*); 240 min.	Long processing may improve exhaustive recovery but increases energy use and volatile-loss risk.	[64]
	Terpenes—α-Pinene: 29.73%; 1,8-cineole: 27.14%; verbenone: 12.81%; borneol: 5.11%.	500 g plant material; 113 °C; 240 min.	Elevated-temperature operation may accelerate recovery but can modify thermolabile volatiles.	[65]
**Combined Clevenger hydrodistillation/steam distillation**	Terpenes—Essential oil yield: 1.50 mL/100 g; 1,8-cineole: 46.71%; α-pinene: 13.83%; camphor: 13.07%.	100 g plant material; 1000 mL distilled water; simultaneous CHD-SD; 180 min.	Combines vapor transport and laboratory collection, but remains thermally intensive.	[9]
**Solar-assisted steam distillation**	Terpenes—Essential oil yield: 17 mL per 2 kg; 1,8-cineole: 22.63%; α-pinene: 43.98%; camphene: 4.58%.	2 kg fresh leaves; two-reflector solar system; 240 min.	May reduce external energy use, but performance depends on irradiation, weather, and process control.	[66]
**Microwave-assisted hydrodistillation (MAHD)**	Terpenes—Essential oil yield: >0.5%; α-pinene: 22.28%; verbenol: 16.49%; 1,8-cineole: 16.26%; geraniol: 5.71%.	Fresh leaves; plant-to-water ratio 1:1 (*w*/*v*); 380 W; 30 min.	Short processing time, but microwave power distribution and scale-up require careful control.	[67]
	Essential oil yield: 1.326%; composition not reported.	Plant material dried for 7 days; water-to-plant ratio 2 mL/g; 600 W; 20 min.	Rapid extraction, although limited compositional reporting prevents selectivity assessment.	[68]
	Terpenes—Essential oil yield: 2.85%; α-pinene: 42.03%; 1,8-cineole: 31.73%; camphor: 12.12%.	150 g plant material; 1000 W; 15 min.	High-intensity treatment is rapid, but local overheating and equipment scale remain concerns.	[69]
**DES-based extraction**	Terpenes—α-Pinene: 9.6%; camphor: 11.2%; rosmarinic acid also reported.	20 mg powder; 1 mL glycerol:xylitol:D-fructose DES (3:3:3); 40 °C; 60 min.	Simultaneous recovery of compounds with different polarities is possible, but viscosity and product separation are important constraints.	[70]
	Terpenes—Camphor: 33.3%; 1,8-cineole: 14.2%; α-pinene: 2.1%.	Choline chloride:glycerol (1:2); 20 °C; 72 h.	Mild temperature but very long extraction; volatile recovery and DES removal require validation.	[71]
**Enzyme-assisted extraction (EAE)**	Terpenes—Extraction yield: 4.10%; 1,8-cineole: 53.48%; β-pinene: 20.23%; borneol: 5.21%.	100 g powder; 500 mL 1.6% cellulase; 46.5 °C; 1.7 h.	Cell-wall disruption may enhance release under mild conditions, but enzyme cost and process control must be considered.	[72]
	Terpenes—Extraction yield: 1.08%; α-pinene: 29.71%; 1,8-cineole: 18.74%; β-pinene: 2.78%; camphor: 2.65%.	15% NaCl solution (5 mL) with 1 g Viscozyme^®^ L; 50 °C; 1 h.	Short pretreatment, but salt addition, enzyme recovery, and downstream volatile isolation add complexity.	[73]

Note: Extraction yields, phytochemical profiles, and operating conditions were compiled from the original studies. Values from independent publications are presented descriptively and are not directly comparable because of differences in plant origin, sample preparation, solvent composition, solid-to-liquid ratio, extraction time and temperature, analytical procedures, and reporting basis. Accordingly, these values should not be used to establish a direct ranking of extraction technologies.

**Table 3:** Solvent-based, assisted, and emerging approaches reported for the recovery and fractionation of rosemary phenolic compounds.

Approach	Reported Recovery/Profile	Key Conditions	Critical Considerations	Ref.
**Hydrodistillation followed by ethanolic extraction**	Polyphenols—TPC: 24.14 mg GAE/g; essential oil yield: 1.57%.	70 g fresh leaves; 350 mL water; 100 °C, 180 min; subsequent 80% ethanol extraction at 70 °C for 60 min.	Supports sequential valorization, but requires two thermal operations and solvent recovery.	[58]
**Hydrodistillation (HD)**	Carnosol: 22.67 mg/g; carnosic acid: 57.36 mg/g; rosmarinic acid: 14.0 mg/g.	50 g fresh material; 500 mL distilled water; 120 min.	Reported phenolics may remain in or be recovered from processed biomass; the analytical basis should be clearly stated.	[74]
**Steam distillation (SD)**	Carnosol: 45.05 mg/g; carnosic acid: 90.67 mg/g; rosmarinic acid: 32.33 mg/g.	2000 g fresh material; distilled water; 120 min.	Potential for recovering phenolics from distillation streams or residues, but mass allocation between fractions is needed.	[74]
**Microwave-assisted hydrodistillation (MAHD)**	Carnosol: 20.12 mg/g; carnosic acid: 46.81 mg/g; rosmarinic acid: 51.34 mg/g.	500 g fresh material; 500 mL water; 1000 W for 5 min, then 500 W for 30 min; 10 min cooling.	Rapid heating may favor release, but compound stability and energy intensity require evaluation.	[74]
**Soxhlet extraction (SE)**	Rosmarinic acid: 33.49 mg/g; carnosic acid: 2.91 mg/g.	Methanol; 70 °C; 6 h.	Exhaustive and reproducible, but solvent use, long heating, and thermal degradation limit sustainability.	[75]
	TPC: 64.51 mg GAE/g.	10 g leaf powder; 250 mL methanol; 50 °C; 360 min.	Useful as a conventional benchmark, but not readily transferable to food or cosmetic use without solvent removal.	[76]
**Maceration followed by Soxhlet extraction**	Yield: 21.58%; TPC: 34.72 ± 1.65 mg GAE/g DW; TFC: 25.02 ± 1.53 mg QE/g DW.	Methanol maceration for 72 h followed by Soxhlet extraction for 6 h.	Sequential exhaustive extraction increases processing time, solvent use, and energy requirements.	[77]
	Yield: 27.92%; rosmarinic acid: 9.86%; carnosic acid: 17.68%; carnosol: 4.39%.	10 g leaves; 5 g pumice; 300 mL ethanol; Soxhlet extraction.	Food-compatible solvent improves applicability, although heating and solvent recycling remain relevant.	[78]
**Ultrasound-assisted extraction (UAE)**	TPC: 214.1 mg GAE/g; rosmarinic acid: 7.2 ± 0.2 μg/mL; carnosol: 2.6 ± 0.1 μg/mL; carnosic acid: 11.2 ± 0.3 μg/mL.	10 g material; 71.3% ethanol; 225.4 W; 20.3 min.	Shorter extraction and lower bulk temperature, but power density and reactor geometry affect scale-up.	[79]
	TPC: 77.5 mg GAE/g; rosmarinic acid: 10.11 mg/g; carnosic acid: 29.1 mg/g.	Distillation residues; 60% ethanol or acetone; UAE, 10 min.	Effective for valorizing residues; solvent choice determines selectivity and downstream compatibility.	[80]
	TPC: approximately 25 mg GAE/g; TFC: 17.45–25.06 mg/g extract.	Dried leaves; plant-to-solvent ratio 1:50; 400 W; 10 min.	Rapid treatment, but a high solvent-to-solid ratio may offset processing advantages.	[6]
**PEF pretreatment followed by UAE**	TPC: 297 mg GAE/100 g FW.	5 g material; 100 mL 55.19% ethanol; 200 W; 50% amplitude; 12.48 min; PEF pretreatment.	PEF may enhance permeability before UAE, but the added unit operation must justify its energy and equipment requirements.	[81]
**Supercritical fluid extraction (SFE)**	Rosmarinic acid: 3.48 mg/g DW.	15 g powder; CO_2_ at 15 g/min with 15% ethanol; 150 bar; 80 °C.	Co-solvent improves polar-compound recovery, but high-pressure equipment and solvent separation are required.	[82]
	Rosmarinic acid: 3.43 mg/g DW; carnosic acid: 18.9 mg/g DW.	15 g powder; CO_2_ at 15 g/min with ethanol; 150 bar; 80 °C.	Selective recovery is condition-dependent; capital and compression energy must be considered.	[83]
**Sequential SFE/fractionation**	Yield: 380 mg/g; rosmarinic acid, carnosol, carnosic acid, and other phenolics.	Supercritical CO_2_ with ethanol; 10–20 MPa; 25 °C; CO_2_ flow 3.0 mL/min.	Can integrate extraction and fractionation, but process complexity and solvent recycling increase.	[84]
**DES-based extraction**	TPC: 18.5 ± 1.65 mg GAE/g; radical inhibition: 88–92%.	Ammonium lactate:acetate-based DES.	Performance is promising, but toxicity, biodegradability, water content, and solvent recovery require case-specific assessment.	[85]
	Rosmarinic acid: approximately 100 μmol/g DW.	Choline chloride:1,2-propanediol (1:5), 20% water; 80 °C; 30 min.	Water reduces viscosity, but temperature, dilution, and DES reuse influence overall sustainability.	[86]
	Rosmarinic acid: 1.6%; α-pinene and camphor also reported.	20 mg powder; 1 mL glycerol:xylitol:D-fructose DES (3:3:3); 40 °C; 60 min.	Broad recovery may be useful, although selectivity and separation of the recovered compounds remain challenging.	[70]
	Rosmarinic acid: 1.00 ± 0.12 mg/g; carnosol: 2.30 ± 0.18 mg/g; carnosic acid: 17.54 ± 1.88 mg/g.	Ratio 1:20 (*w*/*v*); lactic acid:glucose (5:1) or menthol:lauric acid (2:1); 60 °C; 60 min.	Hydrophilic and hydrophobic DESs provide different selectivity; viscosity and downstream recovery must be compared.	[87]
	Carnosic acid: 14.80 mg/g; carnosol: 18.99 mg/g.	Ratio 1:20 (*w*/*v*); 15% tetrapropylammonium chloride and 55% 1,2-propanediol, diluted to 30%; 84 °C; 129 min.	High temperature and long extraction challenge assumptions of inherent greenness.	[88]
	TPC: 78 mg GAE/g.	Liquid-to-solid ratio 40:1; 50% choline chloride:1,2-propanediol; 65 °C.	High solvent ratio and heating may increase resource use despite good recovery.	[89]
	TPC: 2.21 ± 3.85 mg GAE/g; rosmarinic acid: 9.53 mg/g.	150 mg powder; 2.85 mL 90% choline chloride:1,2-propanediol (1:2); 40 °C.	Reported variability and solvent handling should be considered when interpreting performance.	[90]
	TFC: 0.29%; rosmarinic acid: 3.96%.	4 g powder; 40 mL 50% choline chloride:ethylene glycol (1:3); 25 °C; 15 min.	Mild and rapid conditions, but extraction performance and solvent removal remain formulation-specific.	[91]
**Enzyme-assisted extraction (EAE)**	TPC: 15.2 mg GAE/g.	Dried leaves; 20 mL buffer with 25 mg pectinolytic enzyme; 1 h.	Mild processing can assist cell-wall disruption, but enzyme cost, buffer use, and inactivation must be considered.	[92]
	Rosmarinic acid: 13.97 mg/g.	Water-to-sample ratio 28.69 mL/g; 2.56% Cellulase A; 36.6 °C; 4.63 h.	Aqueous processing favors compatibility, although long treatment and water removal may limit efficiency.	[93]

Note: Extraction yields, phytochemical profiles, and operating conditions were compiled from the original studies. Values from independent publications are presented descriptively and are not directly comparable because of differences in plant origin, drying and sample preparation, particle size, solvent composition, solid-to-liquid ratio, extraction time and temperature, analytical procedures, and reporting basis. Accordingly, these values should not be used to establish a direct ranking of extraction technologies. **DW**, dry weight; **FW**, fresh weight; **GAE**, gallic acid equivalents; **QE**, quercetin equivalents; **TPC**, total phenolic content; **TFC**, total flavonoid content.

Comparative analysis of these methods reveals that no single strategy is universally superior, as process efficiency depends on both the chemical nature of the target metabolites and the operating conditions employed during extraction. The reported methodologies can be grouped into three main categories: traditional distillation methods, solvent-based techniques, and assisted or emerging technologies that aim to improve performance or reduce the environmental impact of the process.

Consequently, the selection of an integrated extraction process should begin with the polarity, localization, and thermal stability of the target metabolites. Pretreatments that improve tissue permeability may be combined with selective extraction techniques when mass transfer is limiting; however, unnecessary processing steps can increase energy use, compound degradation, and operating costs. Thus, process integration should be guided by the target phytochemical profile rather than by extraction yield alone.

### 4.2. Distillation Methods for the Recovery of Volatile Compounds

One of the most widely used approaches for obtaining volatile compounds from rosemary is hydrodistillation. This technique, based on the volatilization of aromatic compounds through heating in the presence of water, has been widely employed due to its simplicity and low operating cost. Several studies reported in Table 2 show that the yield of essential oil obtained by hydrodistillation can vary considerably depending on factors such as extraction time, the proportion of water used, and the physiological state of the plant. For example, Gölükcü et al. [56] reported a yield close to 1.8%, with a composition dominated by 1,8-cineole (60.96%) when using a Clevenger system for 120 min. Similarly, Oussaid et al. [60] reported essential oils rich in 1,8-cineole and camphor under prolonged distillation conditions.

These variations in chemical composition and yield can be largely attributed to the influence of environmental and agronomic factors that affect the biosynthesis of secondary metabolites in rosemary. Rathore et al. [59], for example, observed significant seasonal variations in the essential oil composition, with higher concentrations of 1,8-cineole during the rainy season and higher levels of α-pinene during the summer [59]. These results suggest that the phytochemical composition of rosemary is closely related to environmental conditions and the physiological state of the plant, which should be considered when comparing results between different studies.

When comparing the different studies reported for hydrodistillation, it is observed that the essential oil yield can vary depending on seemingly simple operational variables, such as sample size or the proportion of water used during the process. For example, Yeddes et al. [57] reported a yield close to 2.85% using dried leaves and prolonged extraction times, while other studies show lower yields when using fresh plant material or shorter distillation times [57]. This behavior suggests that the partial removal of intracellular water during drying may favor the release of volatile compounds by reducing diffusional resistance within the plant matrix.

Additionally, the chemical composition of the essential oil obtained by hydrodistillation can be affected by the duration of the process. During the early stages of distillation, more volatile compounds, such as 1,8-cineole and α-pinene, are typically recovered, while in later stages, less volatile compounds or products derived from thermal transformations may appear. Therefore, optimizing the distillation time is crucial for obtaining essential oils with specific chemical profiles.

A technique closely related to hydrodistillation is steam distillation, in which steam is generated externally and passed through the plant material. This procedure reduces direct contact with water and can favor the recovery of volatile compounds sensitive to hydrolysis. Khalil and Hassan [62] reported essential oils with compositions dominated by 1,8-cineole (34.25%) and α-pinene (20.98%) using this method [62]. However, the overall efficiency of this technique continues to depend on operational factors similar to those of hydrodistillation, such as the plant-to-water ratio and distillation time.

Distillation methods may be combined with microwave heating or enzymatic pretreatment when faster release of essential oils is required. These combinations can shorten processing time and improve tissue disruption, but excessive heating or prolonged pretreatment may alter the volatile profile and increase energy or enzyme requirements. Their use is therefore most appropriate when essential oil recovery and aroma preservation are prioritized over the extraction of non-volatile phenolics.

### 4.3. Solvent-Based Methods for the Recovery of Phenolic Compounds

While distillation techniques are particularly efficient for recovering volatile compounds, their capacity to extract phenolic metabolites is limited. This difference is mainly explained by the chemical nature of the compounds present in rosemary. Rosemary volatiles include hydrocarbon monoterpenes, such as α-pinene and β-pinene, and oxygenated monoterpenes, such as the cyclic ether 1,8-cineole. These compounds have relatively nonpolar structures that favor their volatilization during distillation. In contrast, the polarity of rosemary phenolic compounds varies according to their functional groups. Rosmarinic acid contains phenolic hydroxyl, ester, and carboxylic acid groups, whereas carnosic acid contains a catechol moiety and a carboxylic acid group. Carnosol is a phenolic diterpene lactone and does not contain a free carboxylic acid group. Consequently, rosmarinic acid is generally recovered using polar solvents, while the more lipophilic carnosic acid and carnosol show greater affinity for less polar solvents or supercritical CO_2_-based processes [94].

In addition to polarity, the molecular size and thermal stability of metabolites also influence their behavior during the extraction process. Monoterpenes have relatively small structures and high volatility, which facilitates their recovery through distillation. Conversely, phenolic diterpenes such as carnosic acid have more complex structures and lower volatility, making their recovery difficult using methods based solely on heating. In this regard, solvent selection plays a fundamental role in the efficiency of the extraction process. Polar solvents, such as methanol or ethanol, have a high capacity to form hydrogen bonds with functional groups present in phenolic compounds, thus promoting their solubilization. In contrast, less polar solvents may be more suitable for the selective extraction of terpenes. For this reason, some studies employ hydroalcoholic mixtures that allow for the simultaneous recovery of metabolites of different polarities.

Representative operating conditions and phenolic recovery outcomes reported for solvent-based methods are summarized in Table 3. In this regard, techniques such as Soxhlet extraction have proven highly efficient for the recovery of phenolic compounds from rosemary. Sharma et al. [75] reported significant concentrations of rosmarinic acid (33,491.33 ± 86.29 µg/g) using methanol as a solvent in a Soxhlet system operated for several hours. Similarly, Zeroual et al. [77] reported high contents of phenolic compounds (34.72 ± 1.65 mg gallic acid equivalent/g dried matter) and flavonoids (25.02 ± 1.53 mg quercetin equivalent/g dried matter) combining maceration and Soxhlet extraction. Although these methods offer high yields, they also have certain limitations, such as long extraction times and the intensive use of organic solvents.

Conventional solvent extraction can serve as an initial exhaustive step or as a reference for assessing intensified technologies. Maceration may be followed by Soxhlet extraction when residual compounds must be recovered, although the additional solvent consumption, processing time, and thermal exposure may outweigh the increase in yield. Alternatively, combining solvent extraction with UAE can reduce extraction time, provided that solvent polarity and ultrasound intensity are selected according to the target phenolic compounds.

### 4.4. Energy-Assisted Technologies for the Intensification of Extraction

**Figure 2:**
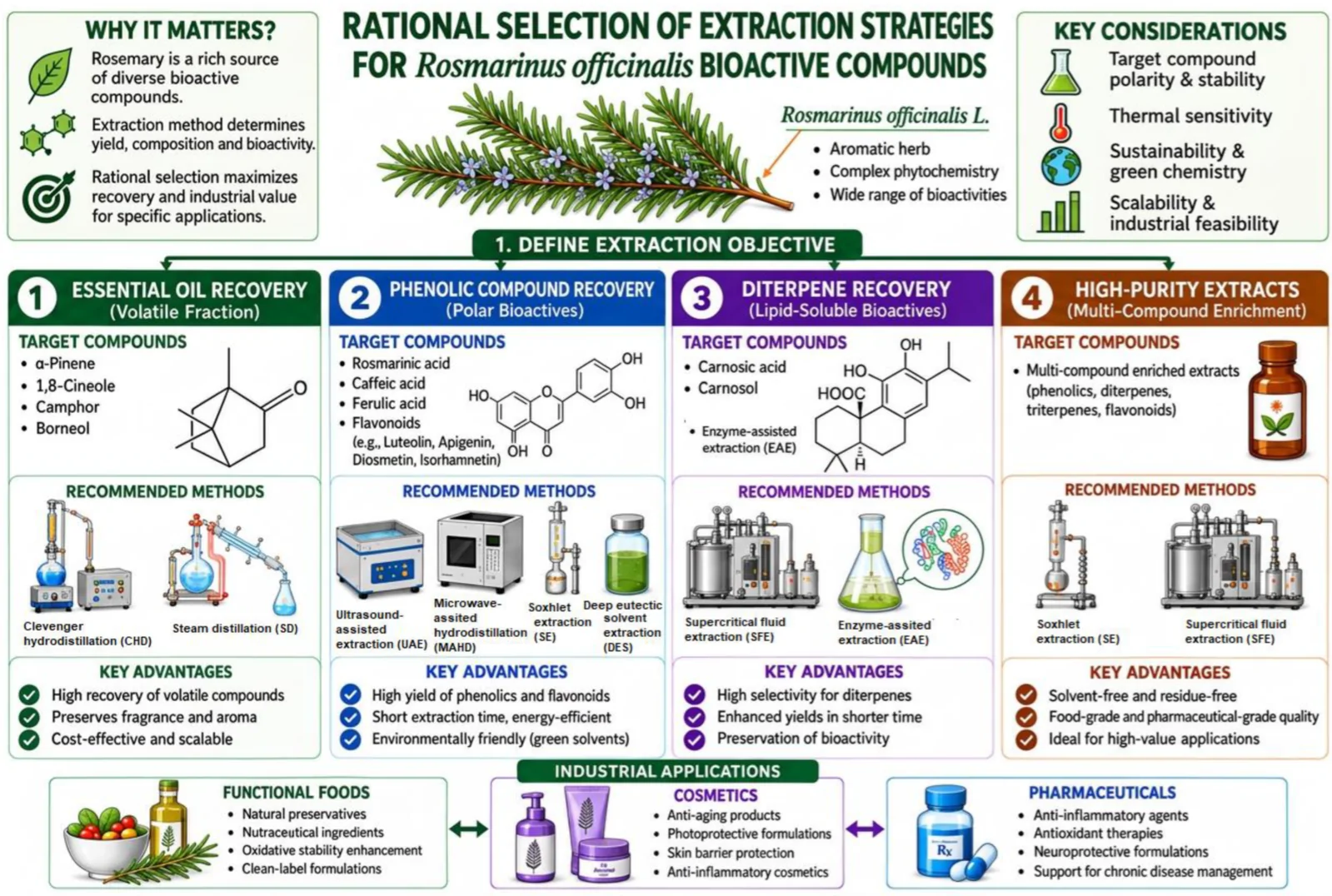
Conceptual map for the selection of extraction strategies aimed at the recovery of specific bioactive compounds from *Rosmarinus officinalis* L.

To overcome these limitations, several energy-assisted techniques have been developed in recent years to improve the efficiency of the extraction process. Among these, ultrasound-assisted extraction and microwave-assisted hydrodistillation stand out. Study-level results for these technologies are presented in Table 2 and Table 3 according to whether the principal recovered fraction consists of volatile or phenolic compounds. The implementation of any of these methodologies, or a combination thereof, along with the availability of the technology, can be geared towards obtaining the metabolites of interest (Figure 2). In the case of ultrasound, the cavitation phenomenon generates microbubbles that collapse near the surface of the plant material, producing mechanical forces capable of breaking down cell walls and facilitating the release of intracellular metabolites. Xie et al. [79,81] reported total phenolic contents exceeding 200 mg GAE/g using this technique. Similarly, Tzima et al. reported a total phenolic content of 297 mg GAE/100 g FW in rosemary by-products following PEF pre-treatment combined with ultrasound-assisted extraction [79,81].

Microwave-assisted hydrodistillation represents another innovative strategy for improving extraction efficiency. In this case, microwaves generate volumetric heating within the plant material, producing internal pressure in the cells and facilitating the release of volatile compounds. Sakar et al. [69] reported essential oil yields close to 2.85% using this technique. In addition to improving yield, this method significantly reduces extraction time compared to conventional hydrodistillation.

Another important factor influencing extraction efficiency is the pretreatment applied to the plant material before the extraction process, as claimed by several authors extracting bioactive compounds and food ingredient from plant-based sources [95,96]. Drying and grinding rosemary can increase the contact surface area between the solvent and the plant matrix, facilitating the diffusion of metabolites into the extraction medium. Elyemni et al. [68] observed that microwave-assisted hydrodistillation (MHD) for rosemary reduces extraction time to 20 min, compared to 180 min for conventional hydrodistillation (HD), making it 9 times faster. Although the total oil yield is similar (~1.32–1.35%), MHD increases the concentration of oxygenated compounds by 1.14% and allows for the identification of a wider variety of compounds.

**Figure 3:**
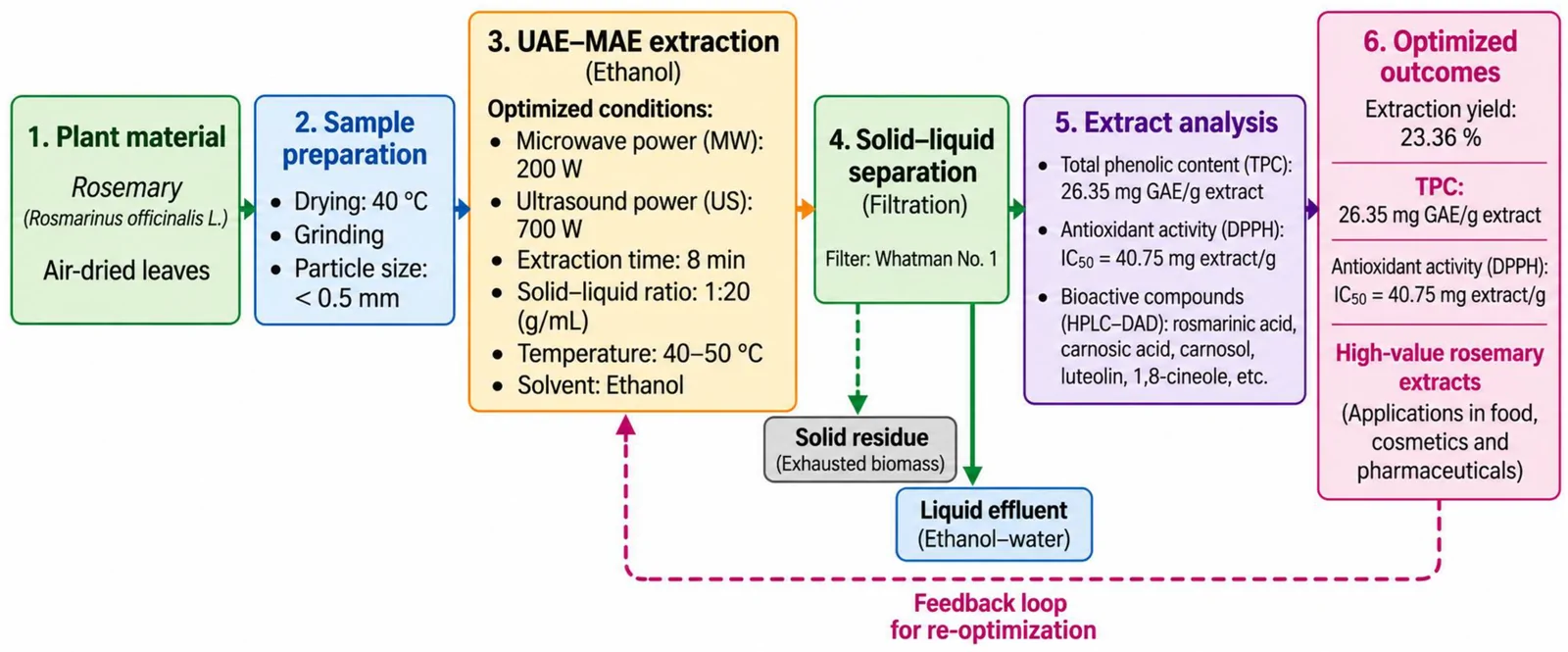
Schematic workflow of the optimized combined ultrasound–microwave-assisted extraction (UAE–MAE) process for *Rosmarinus officinalis* L. Modified from Laina et al. [97] and graphically adapted by the authors to provide an integrated overview of the extraction strategy and its analytical outputs.

Although both microwaves and ultrasound improve extraction efficiency, the physical mechanisms involved in each technique are different. While microwaves generate volumetric heating that increases the internal pressure of plant cells, ultrasound produces cavitation, which involves the formation and collapse of microbubbles that generate intense mechanical forces in the liquid medium. These differences can influence the selectivity of the extraction. In some cases, the combination of both technologies can produce synergistic effects that significantly improve process performance. Laina et al. reported that the sequential application of microwaves and ultrasound yielded extracts with high phenolic content (26.35 mg GAE/g) and high antioxidant activity (Figure 3), demonstrating the potential of integrating different technologies to optimize the metabolite recovery [97].

Energy-assisted technologies are particularly suitable for integration when rapid cell disruption and improved mass transfer are required. UAE may be preceded by PEF or enzymatic treatment, whereas microwave heating can be integrated with hydrodistillation for volatile recovery. Although these combinations may shorten extraction time, excessive energy input can promote local heating, degradation of thermolabile compounds, and additional equipment requirements; therefore, the contribution of each processing step should be evaluated independently.

**Figure 4:**
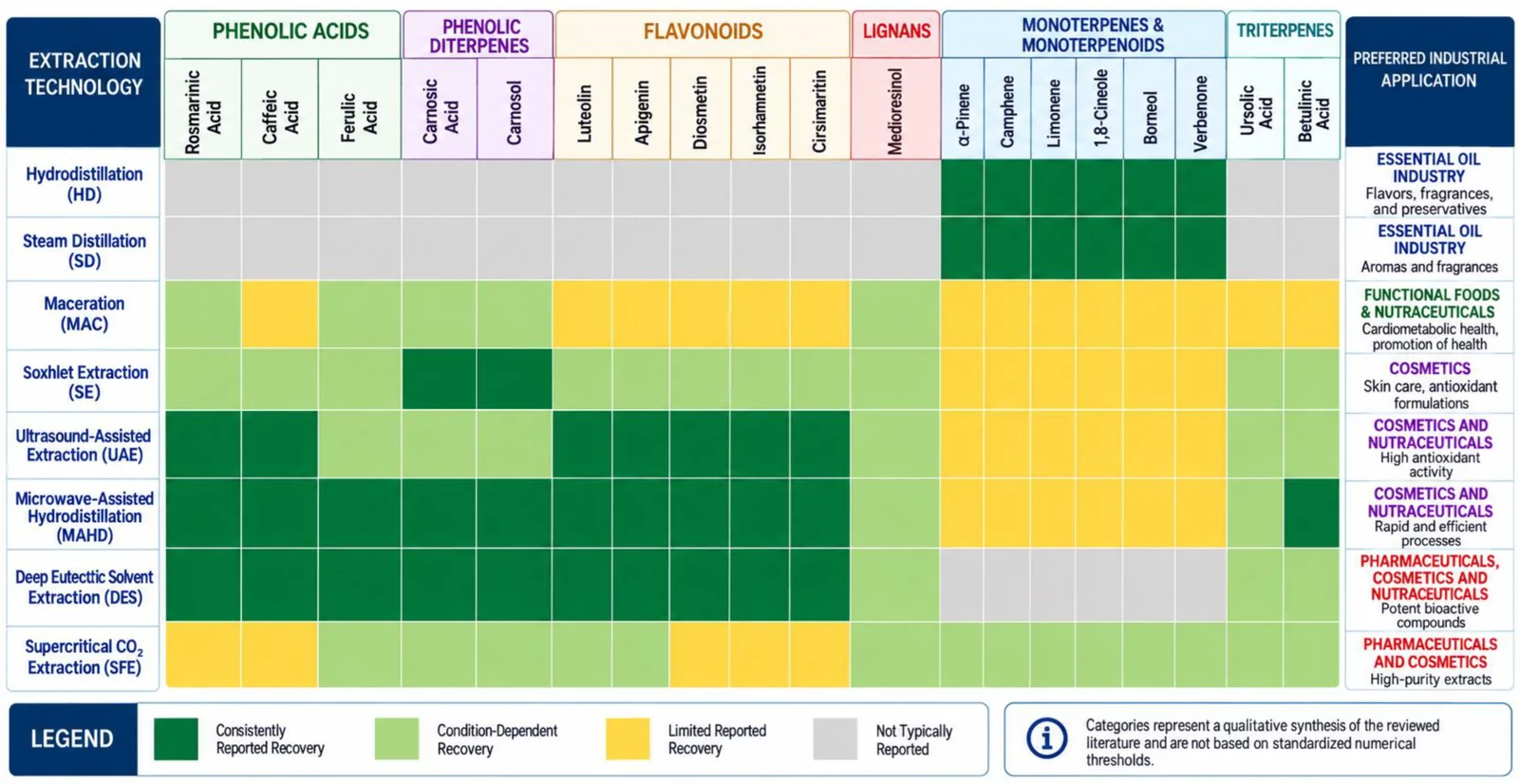
Qualitative evidence map of the reported recovery of the main bioactive metabolite classes of *Rosmarinus officinalis* L. using conventional and emerging extraction technologies. “Consistently Reported Recovery” indicates that the recovery of a metabolite class using a particular technology has been consistently described across the reviewed studies. “Condition-Dependent Recovery” indicates that recovery varies according to solvent composition, operating conditions, or plant material. “Limited Reported Recovery” indicates that the available studies generally report low or restricted recovery. “Not Typically Reported” indicates that the recovery of the corresponding metabolite class using that technology is not commonly described in the available literature.

Efficient recovery of bioactive compounds from rosemary requires the integration of chemical, technological, and industrial factors. Volatile metabolites are preferentially associated with distillation techniques, while phenolic compounds and flavonoids can be recovered using intensified technologies such as ultrasound-assisted extraction or deep eutectic solvent extraction. The transition toward biorefinery processes for *Rosmarinus officinalis* L. valorization is strongly driven by the tunability of deep eutectic solvents (DES), which overcome the selectivity and yield limitations of conventional methods. In the ultrasound-assisted extraction (UAE) of rosemary biomass, the chemical architecture of the DES plays a decisive role in solute affinity; for instance, hydrophilic systems based on choline chloride combined with hydrogen bond donors such as 1,2-propanediol or lactic acid have been reported to increase total phenolic content by up to 220% compared to traditional organic solvents, while simultaneously acting as stabilizing agents for antioxidant capacity [89]. Furthermore, the polarity of the eutectic system dictates the selective fractionation of the matrix: hydrophobic mixtures based on menthol and lauric acid (2:1 molar ratio) exhibit optimal affinity for isolating less polar diterpenes such as carnosic acid and carnosol, whereas polar configurations of lactic acid and glucose (5:1 molar ratio) maximize the recovery of phenolic acids like rosmarinic acid [87]. Thermodynamic optimization of these processes using response surface methodology (RSM) indicates that a controlled water addition between 30% and 50% by weight (wt%), combined with moderate temperatures of 65 °C, mitigates the high inherent viscosity of DES, thereby enhancing acoustic cavitation-driven mass transfer without compromising the structural integrity or the antioxidant and antimicrobial activity of the resulting extracts [89]. In contrast, lipophilic phenolic diterpenes show a greater affinity for microwave-based or supercritical fluid processes. The rational selection of the extraction technology not only determines the yield and preservation of bioactivity but also the viability of downstream applications in functional foods, cosmetics, and pharmaceuticals. This approach allows for establishing a direct relationship between the target metabolite, the extraction strategy, and the added value of the final product [98]. Although extraction technologies are often classified according to their operating principle, their actual performance depends on the chemical nature of the target metabolites. Consequently, the selection of an extraction strategy should not be based solely on the overall process yield, but also on the ability to selectively recover specific compounds. Figure 4 provides a qualitative evidence map of the recovery patterns reported for the main classes of rosemary metabolites. The categories reflect the consistency and variability of the evidence available for each extraction technology and should not be interpreted as standardized extraction efficiencies or as a quantitative ranking. Distillation techniques are predominantly associated with the recovery of volatile constituents, whereas solvent-based and assisted technologies have been reported for a broader range of phenolic compounds. Nevertheless, these patterns remain dependent on solvent composition, operating conditions, plant material, and the analytical approach used in each study. Accordingly, the figure is intended to summarize general trends in the reviewed literature rather than establish the superiority of a particular extraction technology.

### 4.5. Influence of Pretreatments on Extraction Efficiency

Some studies have explored the use of enzymatic treatments to degrade plant cell walls. For instance, Li et al. [72] demonstrated that enzyme-assisted extraction can significantly increase essential oil yield (4.10%) by promoting the disruption of cellular structures. In addition to enzymatic treatments, other physical pretreatments have also been shown to improve extraction efficiency. These include the application of ultrasound prior to the distillation process, the use of pulsed electric fields, or the application of moderate thermal treatments. These strategies aim to increase cell wall permeability or induce microfractures in the plant structure, facilitating the release of metabolites during the extraction stage. The physiological state of the plant material can also influence the efficiency of these pretreatments. Fresh leaves typically contain a higher amount of intracellular water, which can promote heat transfer during microwave-assisted processes. In contrast, dry plant material can facilitate the penetration of organic solvents during the extraction of phenolic compounds.

Pretreatments are most beneficial when structural resistance of the plant tissue limits extraction. PEF can improve membrane permeability before UAE, while cellulases or other hydrolytic enzymes may facilitate the release of compounds retained within the cell wall. Nevertheless, pretreatment may add processing time, water consumption, enzyme costs, or an additional inactivation step, and its benefit should therefore be confirmed against the corresponding extraction process without pretreatment. 

### 4.6. Green Technologies and Emerging Methods

**Figure 5:**
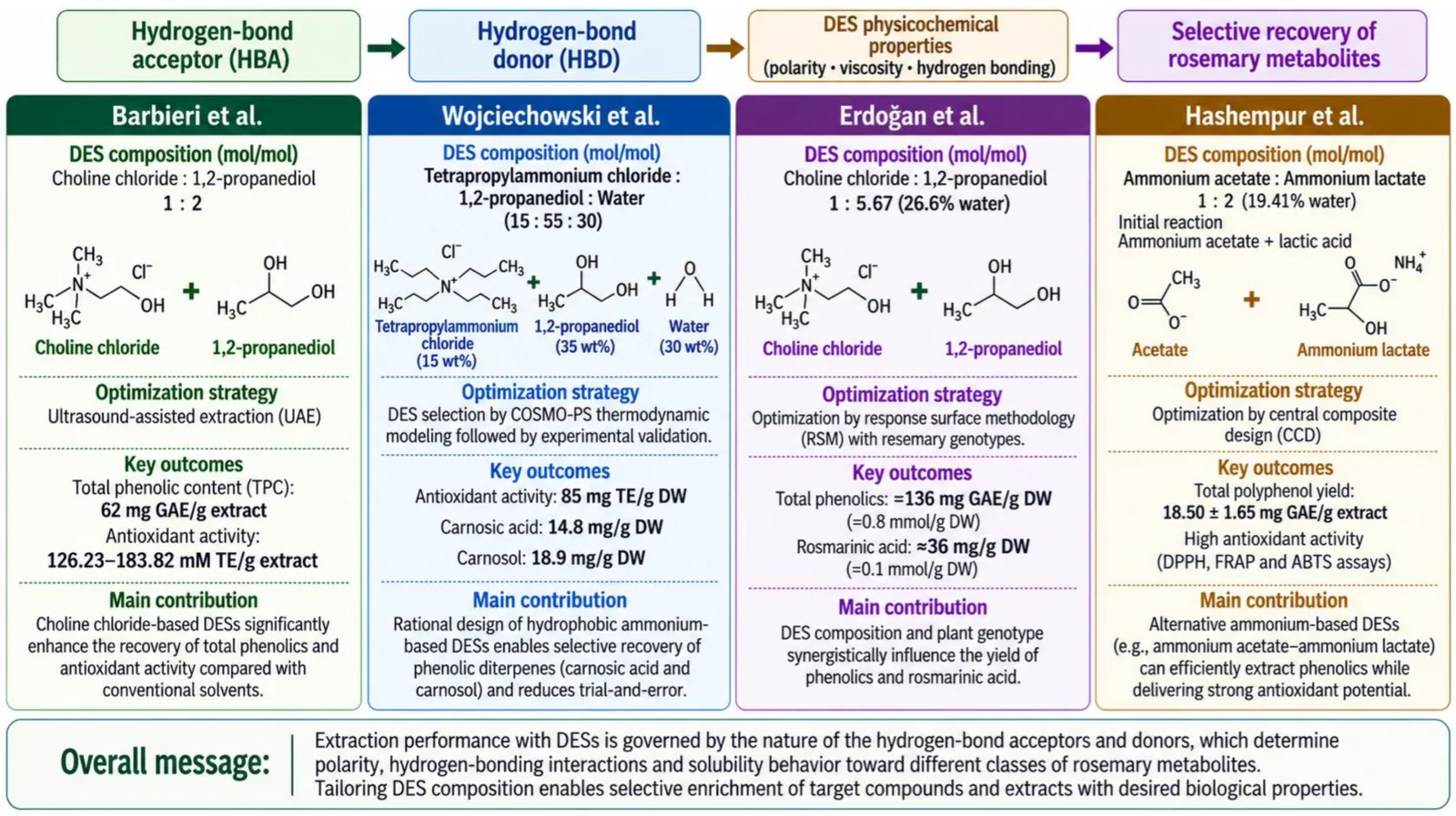
Comparison of representative deep eutectic solvent (DES) systems reported for the extraction of bioactive compounds from *Rosmarinus officinalis* L. The figure summarizes the DES composition, optimization strategy, and principal extraction outcomes of representative studies, illustrating how the combination of hydrogen-bond acceptors and donors governs solvent physicochemical properties and the selective recovery of different classes of rosemary metabolites.

In recent years, considerable attention has been directed toward the development of greener extraction strategies capable of reducing solvent toxicity while maximizing the recovery of rosemary bioactive compounds. Among these approaches, deep eutectic solvents (DESs) have gained attention because of their low volatility, tuneable physicochemical properties, and ability to solubilize phenolic compounds. Nevertheless, DESs should not be considered inherently green, as their toxicity and biodegradability depend on the identity, proportion, and interactions of their individual components. Barbieri et al. demonstrated that choline chloride-based DESs substantially enhanced the extraction of rosemary phenolics compared with conventional solvents, achieving total phenolic contents of up to 62 mg GAE/g extract together with high antioxidant activity (126.23–183.82 mM Trolox equivalents/g) [90]. Likewise, Wojeicchowski et al. integrated the COSMO-RS thermodynamic model with experimental optimization to rationally design DES formulations before laboratory validation, identifying hydrophobic ammonium chloride-based DESs as the most suitable systems for recovering carnosic acid and carnosol. Their optimized extraction reached 85 mg TE/g DW, corresponding to 14.8 mg/g of carnosic acid and 18.9 mg/g of carnosol, while substantially reducing the trial-and-error typically associated with solvent selection [88]. More recently, Erdoğan et al. further demonstrated the versatility of DES-based extraction by combining response surface methodology with different rosemary genotypes and an optimized choline chloride/1,2-propanediol DES (5.67 mol ratio), achieving approximately 0.8 mmol/g DW of total phenolics (≈136 mg GAE/g DW) together with 0.1 mmol/g DW of rosmarinic acid (≈36 mg/g), thereby highlighting the combined influence of plant genotype and solvent composition on extraction selectivity [86]. Similarly, Hashempur et al. optimized an ammonium acetate–ammonium lactate DES using central composite design, obtaining a total polyphenol yield of 18.50 ± 1.65 mg GAE/g while simultaneously producing extracts with remarkable antioxidant and antimicrobial activities [85]. These findings further demonstrate that DES performance depends not only on solvent polarity but also on the nature of the hydrogen-bond donor and acceptor components, which govern solvent–solute interactions and ultimately determine the selective recovery of rosemary phytochemicals. Therefore, the rational design of DES formulations should be guided by the intended application of the extract rather than by extraction yield alone. Moreover, the high viscosity of some DES formulations may increase mixing energy or require water addition, while solvent recovery and removal from the resulting extract can complicate downstream processing. Therefore, their sustainability should be evaluated case by case by considering solvent toxicity and biodegradability, energy and water consumption, recovery and reuse, and subsequent purification requirements, rather than extraction yield alone. Figure 5 summarizes the main DES systems reported for *Rosmarinus officinalis* L., highlighting their composition, extraction strategy, and the principal phytochemical and biological outcomes achieved.

Another emerging green extraction technology is supercritical fluid extraction (SFE) using carbon dioxide (CO_2_), which combines negligible solvent residues with tuneable solvent density and diffusivity, allowing extraction selectivity to be modulated through pressure, temperature, and co-solvent composition. Compared with conventional techniques, SFE offers enhanced preservation of thermolabile compounds while reducing solvent consumption and post-processing requirements. Beyond improving extraction yield, SFE also enables sequential fractionation of phytochemicals with distinct polarities by progressively adjusting extraction conditions. Lefebvre et al. demonstrated this capability by recovering carotenoid-, carnosic acid-, rosmarinic acid-, and chlorophyll-enriched fractions through stepwise modification of CO_2_ pressure and ethanol co-solvent concentration, while simultaneously achieving an extraction yield of 380 mg/g, more than threefold higher than that obtained by ultrasound-assisted extraction (118 mg/g) under the same experimental framework [84].

Importantly, the recovery profile obtained by SFE is compound- and condition-dependent. Supercritical CO_2_ generally favors relatively nonpolar volatile constituents, whereas the incorporation of ethanol as a co-solvent can enhance the recovery of phenolic diterpenes and, to a more variable extent, polar phenolic acids. Consequently, the recovery of individual rosemary metabolites depends strongly on pressure, temperature, co-solvent composition, and the physicochemical properties of the target compounds [99,100].

Beyond improving extraction yield, recent studies have highlighted the ability of SFE to selectively recover different classes of rosemary phytochemicals. Boufetacha et al. integrated an online supercritical fluid extraction-supercritical fluid chromatography (SFE-SFC) platform that enabled sequential recovery of phenolic acids and diterpenes. By operating at 150 bar, 80 °C, and using 15% ethanol as a polar co-solvent, the extraction time decreased from 7.5 to 5.1 min (32% reduction), while the cumulative recovery of rosmarinic acid increased from 3.43 to 5.78 mg/g dry matter, illustrating how process optimization can simultaneously improve efficiency and metabolite recovery [82].

Comparative studies at different processing scales further support the potential advantages of SFE over conventional extraction methods. Carvalho et al. reported that supercritical CO_2_ extraction performed between 100 and 300 bar yielded up to 5.0% extract, whereas conventional hydrodistillation reached only 1.8%, representing an approximately 177% increase in global extraction efficiency while better preserving the native phytochemical profile of Rosemary [100]. Likewise, Visentín et al. demonstrated that a secondary supercritical fractionation step (300 bar, 50 °C) enabled the production of fractions containing up to 33% carnosic acid, highlighting the potential of SFE not only as an extraction technique but also as an efficient downstream purification strategy for obtaining high-value diterpenes [101].

Recent evidence further reinforces the versatility of SFE. Ghedira et al. optimized a multistage supercritical CO_2_ process incorporating ethanol as a co-solvent and reported 115.07 mg GAE/g of total phenolics, substantially higher than those obtained by hydrodistillation (75.23 mg GAE/g) and Soxhlet extraction with ethanol (88.12 mg GAE/g). The supercritical extracts also contained 16–19 mg QE/g of flavonoids and exhibited strong antioxidant activity (IC_50_ = 1.8–5.5 μg/mL), while achieving an exceptionally high concentration of carnosic acid (306.3 mg/g) under 270 bar with ethanol as co-solvent, compared with only 29.6 mg/g in the same process without co-solvent and 5.09 mg/g in ethanolic Soxhlet extracts. Interestingly, Soxhlet extraction remained the most effective approach for recovering rosmarinic acid (39.3 μg/g), indicating that solvent polarity and extraction mechanism can be tuned to preferentially enrich different phenolic subclasses [102].

Emerging technologies may also be integrated to combine extraction selectivity with subsequent fractionation. UAE can reduce the mass-transfer limitations associated with viscous DES, although solvent recovery, dilution requirements, and downstream purification remain important constraints. Likewise, SFE may be coupled with co-solvent addition or SFC to recover and fractionate compounds of different polarities, but this benefit must be balanced against high-pressure operation, equipment complexity, and scale-up costs.

**Figure 6:**
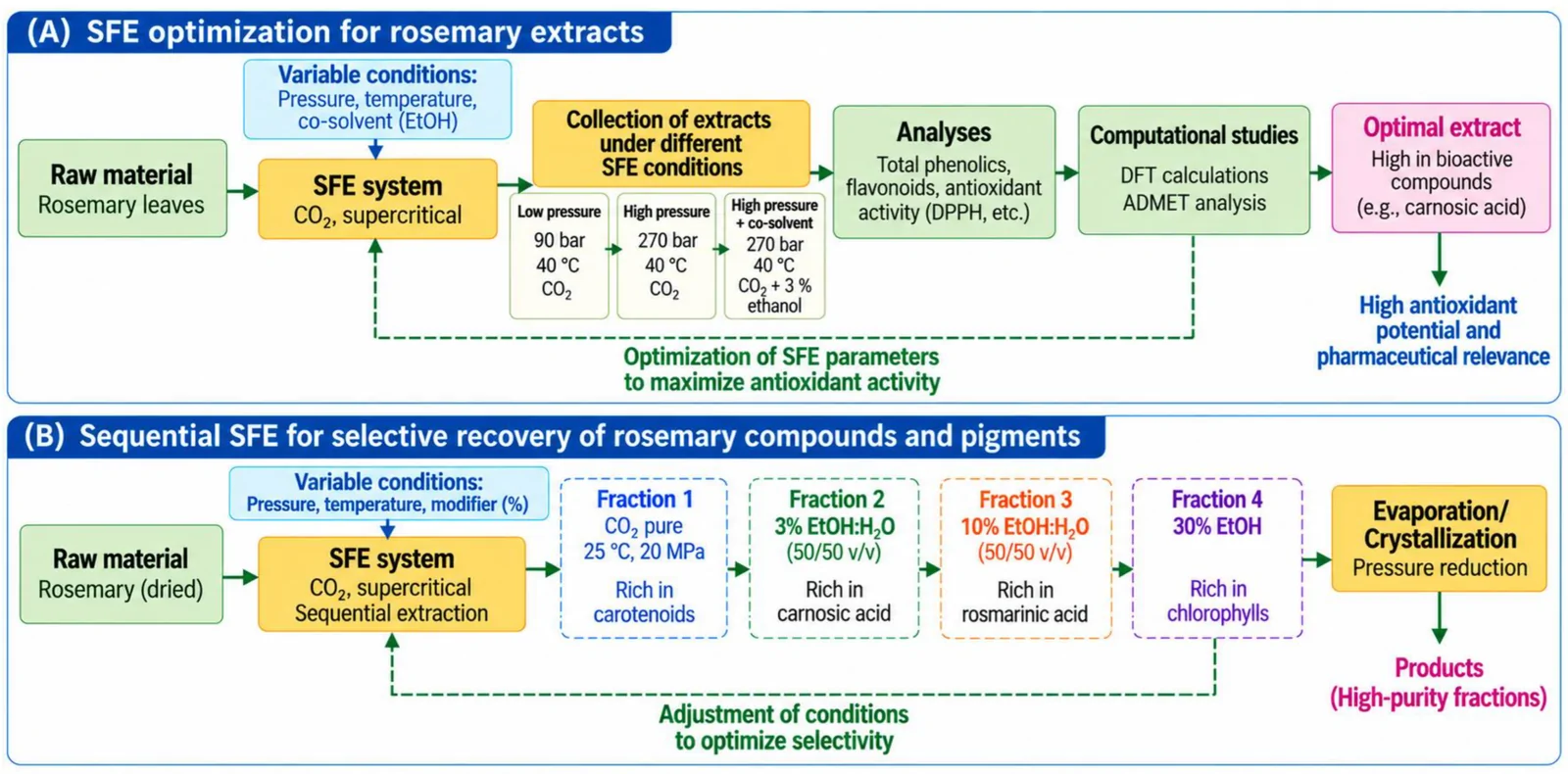
Comparison of two supercritical fluid extraction (SFE) strategies applied to *Rosmarinus officinalis* L. (**A**) Multistage SFE process optimized by Ghedira et al. for maximizing the recovery of antioxidant compounds through modulation of pressure and ethanol co-solvent, followed by physicochemical and computational characterization of the extracts. (**B**) Sequential SFE strategy developed by Lefebvre et al., where stepwise modification of extraction conditions enables the selective recovery of carotenoids, carnosic acid, rosmarinic acid, and chlorophyll-rich fractions. Together, both approaches demonstrate the versatility of SFE for either maximizing global extraction efficiency or selectively enriching specific classes of bioactive compounds.

Overall, these studies demonstrate that supercritical fluid extraction has evolved into a highly versatile extraction platform whose performance can be tailored according to the desired objective. As illustrated in Figure 6, SFE can be configured either to maximize the overall recovery of antioxidant phytochemicals through process optimization or to selectively fractionate individual classes of metabolites by sequentially modifying extraction conditions. This operational flexibility, achieved by controlling pressure, temperature, and co-solvent composition, enables the selective enrichment of target compounds while preserving extract quality and reducing downstream purification requirements.

### 4.7. Direct Comparison Between Extraction Technologies

Table 4 complements Table 2 and Table 3 by presenting extraction methods evaluated within individual comparative studies under common or closely comparable experimental conditions. Whereas Table 2 and Table 3 compile descriptive results from independent publications, Table 4 provides a more appropriate basis for within-study comparisons. For example, Wang et al. [103] compared hydrodistillation, enzyme-assisted extraction, ultrasound-assisted extraction, and DES-based extraction and reported the highest yield for the DES system under the evaluated conditions. This result can be explained by the ability of DES to establish multiple intermolecular interactions with phenolic compounds, thus improving their solubilization. Similarly, Ngan et al. compared hydrodistillation, steam distillation, and microwave-assisted extraction, finding that microwave-assisted hydrodistillation produced a higher yield than the conventional methods evaluated within the same study. This increase in yield is mainly attributed to the ability of microwaves to accelerate cell disruption and promote the release of volatile metabolites [104].

Irakli et al. demonstrated that ultrasound-assisted extraction methods can recover higher concentrations of phenolic compounds from rosemary distillation residues (53.91 mg/g of rosmarinic acid) compared to traditional techniques such as Soxhlet (51.40 mg/g of rosmarinic acid). These results highlight the potential of emerging technologies to improve the recovery efficiency of bioactive metabolites, even from residues generated during essential oil distillation [105]. Finally, when evaluating these techniques from an industrial scalability perspective, it is important to consider not only extraction yield but also factors such as equipment cost, energy consumption, process integration, and techno-economic feasibility [106,107]. Distillation techniques continue to be widely used in industry due to their operational simplicity, robustness, and ease of scale-up. However, emerging technologies such as ultrasound- and microwave-assisted extraction have demonstrated considerable potential for industrial implementation, particularly when integrated into continuous processing systems [108]. Likewise, green solvent- and supercritical fluid-based extraction methods offer important environmental advantages by reducing organic solvent consumption and improving extract quality, although their large-scale implementation still requires further optimization, standardization, and comprehensive techno-economic evaluation [106,109].

These comparisons indicate that no single extraction technology is optimal for all rosemary metabolites. Sequential or hybrid processes are justified when the first step facilitates compound release and the second provides greater selectivity or fractionation; however, improvements in recovery should be evaluated together with energy demand, solvent use, processing time, and extract composition. Comparisons should preferably be based on techniques evaluated using the same plant material and analytical basis.

### 4.8. Perspectives and Considerations for the Industrial Selection of Extractive Technologies

Although considerable progress has been made in developing sustainable technologies for the recovery of bioactive metabolites from *Rosmarinus officinalis L*., several scientific and technological challenges remain unresolved. Future research should focus not only on improving extraction efficiency but also on increasing process selectivity, sustainability, scalability, and industrial applicability. Based on the current literature, the following perspectives deserve particular attention:

- **Rational selection of extraction technologies.** Most studies continue to compare extraction methods primarily based on overall extraction yield. However, *Rosmarinus officinalis* L. contains chemically diverse metabolites whose extraction behavior differs markedly according to their polarity, volatility, and thermal stability. Highly volatile monoterpenes such as α-pinene and 1,8-cineole are efficiently recovered by distillation, whereas hydrophilic phenolic acids (e.g., rosmarinic acid) and lipophilic phenolic diterpenes (e.g., carnosic acid and carnosol) require fundamentally different extraction environments [110]. Future studies should therefore prioritize metabolite-oriented extraction strategies rather than maximizing crude extract recovery, establishing standardized workflows specifically designed for each phytochemical family [111,112].
- **Integration of pretreatment and extraction processes.** Despite the recognized influence of pretreatment on mass transfer, relatively few studies have systematically investigated its effect on rosemary phytochemicals [113]. Since glandular trichomes are the primary storage sites for volatile terpenes, whereas phenolic compounds are mainly localized within intracellular tissues, pretreatment strategies should be adapted according to the target metabolites [53,114]. Future research should evaluate how drying conditions, particle size reduction, enzymatic disruption, pulsed electric fields, or ultrasound pretreatment affect not only extraction yield but also the preservation of oxidation-sensitive compounds such as carnosic acid, whose conversion into carnosol may substantially modify the phytochemical profile [115,116,117].
- **Development of intelligent green solvent systems.** Deep eutectic solvents have demonstrated excellent potential for rosemary extraction owing to their tunable polarity and hydrogen-bonding capacity. Nevertheless, solvent selection remains largely empirical. Future studies should rationally design DES formulations according to the distinct chemical characteristics of rosemary metabolites. For example, highly polar systems could preferentially recover rosmarinic acid through hydrogen-bond interactions [118], whereas hydrophobic DESs may improve the selective extraction of lipophilic diterpenes such as carnosic acid and carnosol [119]. The integration of computational tools, including COSMO-RS and molecular simulations, could considerably accelerate solvent design while improving extraction selectivity [88].
- **Artificial intelligence-assisted process design.** The growing availability of experimental datasets generated from different extraction technologies provides an unprecedented opportunity to integrate artificial intelligence (AI) into the rational development of rosemary extraction processes. Machine learning algorithms can identify nonlinear relationships among extraction variables, solvent composition, and phytochemical recovery that are often overlooked by conventional statistical approaches. Combined with computational tools such as COSMO-RS, AI-driven models could accelerate the prediction of optimal DES formulations, extraction conditions, and metabolite selectivity while substantially reducing experimental workload [120]. Beyond process optimization, future developments may incorporate digital twins capable of simulating extraction performance under different processing scenarios, facilitating scale-up, process control, and the production of standardized rosemary extracts [121,122,123].
- **Integration of extraction and downstream purification.** Current studies largely emphasize extraction efficiency, whereas considerably less attention has been devoted to obtaining purified fractions enriched in individual rosemary metabolites. This limitation is particularly relevant because cosmetic, pharmaceutical, and functional food applications frequently require standardized concentrations of compounds such as carnosic acid or rosmarinic acid rather than crude extracts. Future work should integrate extraction with downstream purification strategies including membrane separation, adsorption processes, preparative chromatography, or hybrid systems capable of selectively isolating phenolic diterpenes from phenolic acids while preserving their biological activity [124,125].
- **Scale-up and techno-economic assessment.** Although supercritical fluid extraction, ultrasound-assisted extraction, microwave-assisted extraction, and deep eutectic solvent extraction have demonstrated excellent laboratory performance, their industrial feasibility remains poorly documented. Future investigations should evaluate solvent recycling, energy consumption, process intensification, and extraction costs while considering the specific requirements of rosemary-derived products [126]. In particular, the economic feasibility of producing standardized extracts enriched in carnosic acid, rosmarinic acid, or essential oil fractions should be compared with that of conventional crude extracts intended for cosmetic and functional food formulations.
- **Toward an integrated rosemary biorefinery.** Rather than targeting a single compound, rosemary processing could adopt a sequential approach for valorizing its chemically diverse metabolite families [127,128]. Volatile monoterpenes could first be recovered by steam or hydrodistillation [129], followed by selective extraction of phenolic diterpenes using supercritical CO_2_ or hydrophobic DESs [130,131], and subsequent recovery of rosmarinic acid and flavonoids from the remaining biomass using hydrophilic solvents [80]. However, the sequence of these operations is critical, because prolonged thermal exposure may alter thermolabile phenolics, while the moisture retained after distillation may increase drying requirements or solvent consumption during subsequent extraction. Therefore, the composition and recovery achieved at each stage should be evaluated before considering these processes technically complementary [5,127,129].

Demonstrating the feasibility of this strategy would require a complete mass balance tracking the initial biomass, essential oil, hydrosol, phenolic extracts, purified fractions, recovered solvents, and final solid residues. Solvent and water recycling may reduce environmental burdens but can introduce additional energy requirements and affect solvent purity, whereas downstream operations such as membrane separation, adsorption, or chromatography may improve extract quality at the cost of greater process complexity [125]. Residual biomass could potentially be directed toward composting, bioenergy, or other value-added uses, provided that solvent residues and biological safety are assessed. Therefore, life-cycle and techno-economic analyses remain necessary before describing the proposed scheme as an industrially sustainable biorefinery [129,132].

Taken together, these perspectives indicate that future advances in rosemary extraction should move beyond the optimization of individual processing parameters toward the rational design of integrated extraction platforms tailored to specific phytochemical targets and end-use applications. This transition is particularly evident for DESs, whose extraction performance cannot be generalized across all classes of rosemary metabolites because it is intrinsically governed by their physicochemical properties. Rather than continuing the empirical screening of new solvent combinations, future research should focus on developing task-specific DES formulations according to the chemistry of the target compounds. For example, highly polar DESs enriched in hydrogen-bond donors are expected to preferentially recover phenolic acids such as rosmarinic acid through strong hydrogen-bonding interactions, whereas hydrophobic DES systems are better suited for extracting lipophilic phenolic diterpenes such as carnosic acid and carnosol. A deeper understanding of these solvent–solute interactions, supported by computational approaches such as COSMO-RS and molecular simulations, will be essential for establishing predictive extraction platforms for *Rosmarinus officinalis* L. rather than relying on trial-and-error solvent selection [88,103,118,119].

At an industrial scale, integrating extraction technologies is appropriate only when the additional step provides a measurable improvement in yield, selectivity, stability, or downstream processing. A technically effective combination may still be unsuitable if it requires difficult solvent recovery, high water or energy consumption, specialized equipment, or costly process control. Future comparisons should therefore incorporate economic assessment, solvent recyclability, scalability, and environmental indicators alongside extraction performance.

Likewise, future studies should place greater emphasis on the long-term physicochemical stability of DES formulations, since changes in water content, viscosity, hydrogen-bond network organization, or component degradation during extraction and storage may alter extraction selectivity, reproducibility, and the stability of oxidation-sensitive metabolites such as carnosic acid [133]. These aspects become particularly relevant when considering solvent recycling, as the industrial feasibility of DES-based processes will depend not only on solvent recovery but also on maintaining extraction performance over successive cycles while minimizing operational costs [134,135]. Ultimately, the next generation of rosemary extraction technologies should shift from developing universally applicable green solvents toward engineering robust, recyclable, and metabolite-oriented DES systems capable of producing standardized extracts tailored to cosmetic, functional food, and pharmaceutical applications [105,136].

## 5. Applications of Rosemary (*Rosmarinus officinalis* L.) in Functional Foods and Dermatology

**Figure 7:**
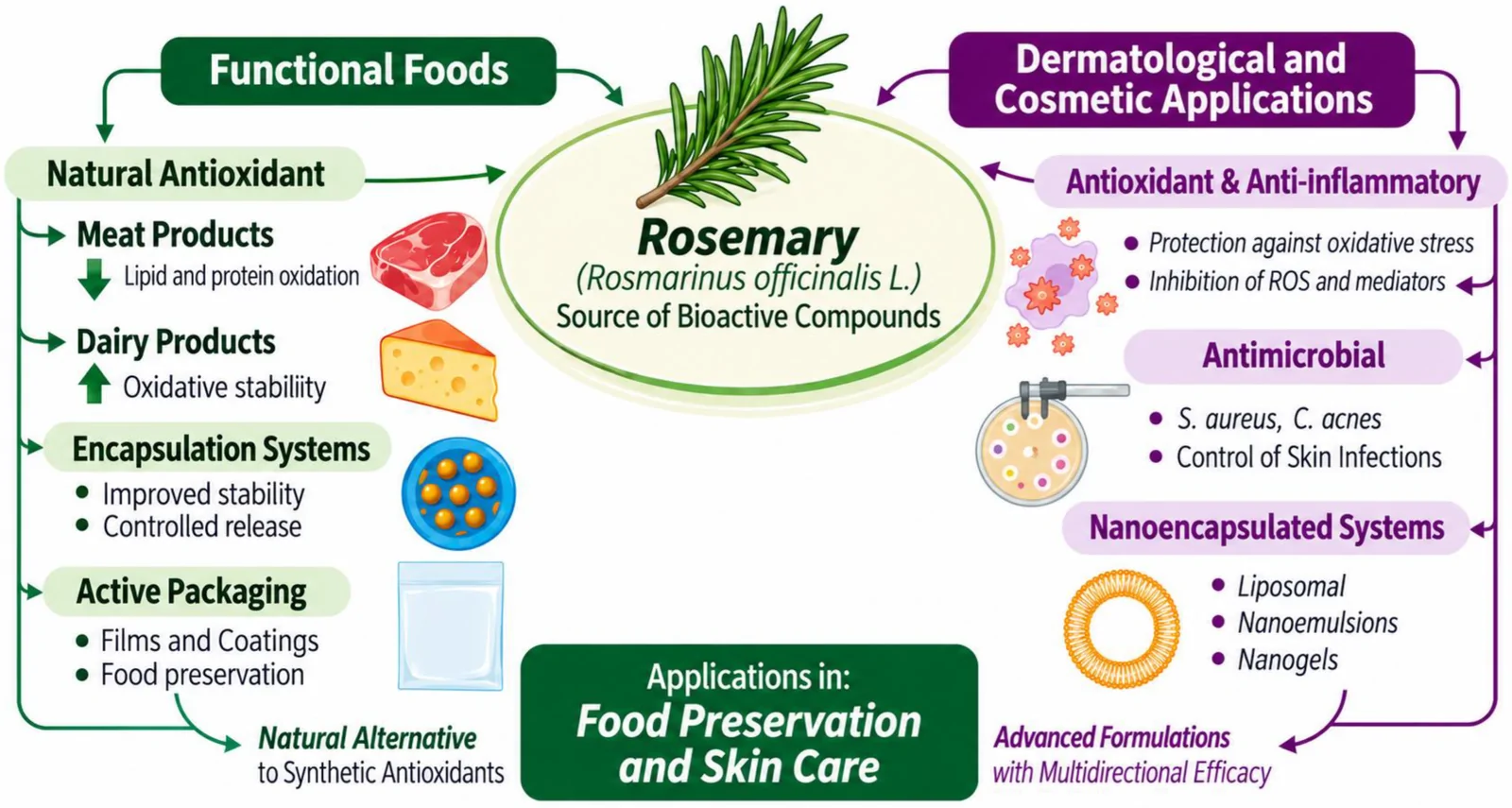
Conceptual map summarizing the main bioactive compounds present in *Rosmarinus officinalis* L. and their emerging applications in functional foods and dermatology. Arrows indicate the direction of the reported antioxidant effect of rosemary-derived compounds in food systems: ↓ denotes reduced lipid and protein oxidation, whereas ↑ denotes increased oxidative stability.

Rosemary (*Rosmarinus officinalis* L.) has become established as a significant source of naturally occurring bioactive compounds, primarily due to its high content of phenolic diterpenes (carnosic acid and carnosol) and hydrophilic phenolic compounds such as rosmarinic acid. These metabolites and rosemary-derived extracts have been associated with antioxidant, antimicrobial, and anti-inflammatory activities. This evidence has supported the investigation of rosemary-derived ingredients in food matrices and, increasingly, in dermatological and cosmetic formulations, as illustrated in Figure 7. These compounds have been explored as natural antioxidants in food matrices, encapsulation systems, and active packaging materials. In parallel, in dermatology and cosmetics, rosemary extracts and selected isolated metabolites are being investigated for their ability to protect the skin against oxidative stress and inflammation, particularly through advanced nanoencapsulation systems.

In the field of functional foods, rosemary extracts have been evaluated as natural alternatives to synthetic antioxidants to improve the oxidative stability of products susceptible to lipid degradation. Studies using rosemary extracts and rosemary essential oil in meat and milk products have reported that the addition of rosemary extracts significantly reduces lipid and protein oxidation during storage, delaying the onset of rancidity and preserving key sensory attributes such as color and aroma [137,138,139]. Similar results have been reported in dairy matrices, where fortification with rosemary extracts improved the oxidative stability of milk, cheeses, and yogurts, although it has been noted that high concentrations can affect sensory acceptability, highlighting the importance of controlled dosage [140,141,142,143].

Because several rosemary-derived antioxidant compounds are susceptible to degradation in the presence of light, oxygen, or elevated temperature, different encapsulation strategies have been investigated to protect their biological activity and facilitate their incorporation into functional foods. In this regard, encapsulation of rosemary extracts in polysaccharide- or protein-based systems has improved their stability during processing and storage [144,145,146]. These approaches have also been extended to the development of active packaging materials, where films and coatings incorporating rosemary extract have demonstrated antioxidant and antimicrobial capacity, contributing to the preservation of perishable foods [147,148,149]. In parallel, interest in rosemary has grown considerably in the dermatological and cosmetic fields, particularly for its potential to protect the skin against oxidative stress and inflammation. Antioxidant effects in skin cell models have been described for rosmarinic acid and phenolic diterpenes, which are identified in rosemary extracts and oils. This is relevant considering the role of oxidative stress in skin aging and various inflammatory dermatoses [27,150,151,152]. Rosemary essential oil and selected rosemary extracts have shown antimicrobial activity against microorganisms involved in skin disorders, such as *Staphylococcus aureus* and *Cutibacterium acnes*, supporting their potential investigation as ingredients in dermocosmetic products intended for acne-prone skin [153,154,155].

Additionally, topical formulations incorporating rosemary extracts or purified fractions have demonstrated anti-inflammatory and photoprotective effects, primarily attributed to the inhibition of pro-inflammatory mediators and the partial absorption of UV radiation. Anti-inflammatory or photoprotective effects have been reported in studies evaluating rosemary-derived extracts or topical formulations. However, the type and strength of the evidence vary according to the tested material and experimental model [156,157,158,159].

An emerging and still underexplored application of rosemary is its potential use in regenerative wound care and scar modulation. Recent preclinical evidence showed that a topical ethanolic rosemary extract accelerated re-epithelialization and reduced fibrosis in murine wound models, while experiments with isolated carnosic acid identified activation of TRPA1 in cutaneous sensory neurons as a potential mechanism promoting tissue regeneration [27]. Complementary in vitro studies with rosemary fractions have also reported enhanced fibroblast migration together with antioxidant and anti-inflammatory effects [3]. Nevertheless, these findings should be considered preliminary, as standardized extract composition, effective dose, cutaneous safety, and clinical efficacy in humans remain to be established.

In this context, the nanoencapsulation of rosemary extracts and essential oils in liposomes, nanogels, and nanoemulsions has been investigated as an effective strategy to improve its stability, cutaneous bioavailability, and controlled release, overcoming limitations such as its low aqueous solubility and susceptibility to degradation [160,161,162,163,164,165]. Taken together, the available evidence suggests that rosemary-derived extracts and compounds represent promising bioactive ingredients for functional foods and dermatological applications. Nevertheless, the strength of this evidence varies among isolated compounds, crude or standardized extracts, and complete formulations. However, despite the growing number of studies, the application of purified or encapsulated rosemary extracts still requires further research to evaluate their long-term stability, sensory impact in complex matrices, and in vivo efficacy, particularly in the context of advanced topical formulations.

## 6. Concluding Remarks

*Rosmarinus officinalis* L. is a valuable source of phenolic acids, phenolic diterpenes, flavonoids, monoterpenes, and triterpenes with potential applications in food, cosmetic, and pharmaceutical products. Nevertheless, the strength of the available evidence varies considerably among isolated compounds, rosemary extracts, essential oils, and formulated products. Biological activities demonstrated for individual metabolites should therefore not be directly extrapolated to complete extracts or commercial applications without appropriate experimental validation.

The reviewed literature also indicates that no extraction technology is universally superior. Reported performance depends on the plant material, target metabolites, solvent composition, solid-to-liquid ratio, operating conditions, analytical method, and reporting basis. Consequently, results obtained in independent studies should be interpreted descriptively rather than used to establish direct rankings. Although UAE, microwave-assisted extraction, SFE, and DES-based systems offer relevant opportunities for process intensification and selective recovery, their sustainability and industrial feasibility cannot be inferred solely from extraction yield. Energy and water consumption, solvent toxicity and recyclability, equipment requirements, downstream purification, process scale, and residual biomass management must also be considered.

Future research should prioritize standardized reporting, direct comparisons under equivalent experimental conditions, complete mass balances, solvent-recycling studies, and the integration of life-cycle and techno-economic assessments. Additional in vivo, stability, safety, and clinical studies are also needed to support advanced food and dermatological applications. Thus, the most appropriate extraction and valorization strategy should be selected according to the target metabolite, desired product specifications, available processing infrastructure, and intended application rather than assuming the general superiority of a particular technology.

## Figures and Tables

**Table 1:** Main secondary metabolites present in rosemary (*Rosmarinus officinalis L*.), their biological activities and potential applications in functional foods and cosmetic products.

Compound	Chemical Family	Main Biological Activity	Reported or Potential Application in Functional Foods	Reported or Potential Application in Cosmetics	Refs.
**Rosmarinic acid**	Phenolic acid	Antioxidant, anti-inflammatory, antimicrobial	Natural preservative; protection against lipid oxidation; shelf-life extension	Antioxidant, anti-aging, protection against UV-induced oxidative stress	[24] ^a^
**Carnosic acid**	Phenolic diterpene	Potent antioxidant, neuroprotective	Natural preservative in oils and fatty foods	Antioxidant, photoprotective, anti-aging	[25,26] ^b^
**Carnosol**	Phenolic diterpene lactone	Antioxidant, anti-inflammatory, antimicrobial	Oxidative stabilizer in foods	Dermal protective agent, antioxidant	[25,27] ^b,c^
**Caffeic acid**	Phenolic acid	Antioxidant, antimicrobial, anti-inflammatory, free radical scavenger	Nutraceutical ingredient; prevention of oxidation	Anti-aging, cellular protective agent	[28,29] ^a,b^
**Ferulic acid**	Phenolic acid	Antioxidant, antimicrobial, antidiabetic, photoprotective	Natural preservative and stabilizer; supplements against metabolic syndrome	UV-protective agent, complementary depigmenting agent	[30] ^a,b,c^
**Luteolin**	Flavonoid	Antioxidant, anti-inflammatory, antimicrobial, anti-cancer, anti-viral, anti-diabetic	Cardiovascular protection; nutraceutical; preservation, anti-diabetic products	Regulation of aging of skin and inflammation	[31] ^b,c^
**Apigenin**	Flavonoid	Antioxidant, anti-inflammatory, anti-cancer, anti-viral	Functional ingredient with cardioprotective potential	Skin-soothing agent, dermal protective agent, anti-aging, photoprotective	[32,33] ^a,b,c^
**Diosmetin**	Flavonoid	Antioxidant, anti-inflammatory, antimicrobial, vasoprotective	Potential nutraceutical	Anti-inflammatory and cutaneous vascular protective agent	[34] ^c^
**Cirsimaritin**	Flavonoid	Antioxidant, anti-inflammatory, anti-cancer, antiparasitic, antimicrobial	Natural food preservation	Antioxidant and antimicrobial agent	[35] ^c^
**Isorhamnetin**	Flavonol	Antioxidant, anti-inflammatory, antidiabetic	Nutraceutical with metabolic potential	Anti-aging and cellular protective agent	[36,37] ^a,b^
**Medioresinol**	Lignan	Antioxidant, potential neuroprotective activity	Potential functional ingredient	Potential dermal protective agent	[38] ^a^
**α-Pinene**	Hydrocarbon monoterpene	Antimicrobial, antioxidant, neuroprotective	Natural flavoring agent and preservative; gastroprotective	Skin photoaging, improve percutaneous permeation	[39,40] ^a,b^
**Camphene**	Monoterpene	Antioxidant, anti-cancer, antidiabetic, antiparasitic	Flavoring agent, potential metabolic modulator	Skin penetration enhancers and stabilizers in topical formulations; fragrances and toning products	[41,42] ^a–c^
**Limonene**	Hydrocarbon monoterpene	Antioxidant, anti-inflammatory, antimicrobial	Natural flavoring agent, preservative	Fragrances, hair and dermocosmetic products	[43,44] ^a–c^
**1,8-Cineole**	Oxygenated monoterpene—cyclic ether	Antimicrobial, anti-inflammatory	Natural preservative and flavoring agent	Antiseptic, muscle-relaxing agent	[45,46] ^a,b^
**Borneol**	Oxygenated monoterpene—alcohol	Antioxidant, anti-inflammatory	Potential functional ingredient	Fragrances, soothing and refreshing agent	[47,48] ^a,b^
**Verbenone**	Oxygenated monoterpene—ketone	Antimicrobial, aromatic	Natural flavoring agent	Fragrances and aromatherapeutic cosmetics	[49] ^a^
**Camphor**	Oxygenated monoterpene—ketone	Topical analgesic, antimicrobial, antiviral	Limited use as flavoring agent	Cooling and muscle-care products, antibacterial agent	[42] ^a–c^
**Ursolic acid**	Pentacyclic triterpene	Anti-inflammatory, antioxidant, antimicrobial	Nutraceutical ingredient, preservative	Anti-aging, firming agent, skin cancer prevention	[50,51] ^b,c^
**Betulinic acid**	Pentacyclic triterpene	Antioxidant, antimicrobial, antitumor, anti-inflammatory	Potential functional ingredient	Potential regenerating and protective cosmetics	[52] ^a,b^

^a^ Evidence obtained using an isolated compound; ^b^ evidence obtained using a rosemary extract or essential oil; ^c^ evidence obtained using a rosemary-containing formulation. Applications inferred from compound-level evidence are presented as potential uses and should not be interpreted as demonstrated efficacy of a complete rosemary extract or formulated product.

**Table 4:** Outcomes of extraction methods evaluated within individual comparative studies.

Extraction Method	Optimized Extraction Conditions	Extract Yield (%)	1,8-Cineole (%)	α-Pinene (%)	β-Pinene (%)	Rosmarinic Acid (mg/g DW)	TPC (mg GAE/g DW)	Ref.
**CHD**	Water; 180 min	~0.63	27.55	NR	19.80	NR	NR	[103]
**EAE**	Cellulase pretreatment; 50 °C; 2 h	~0.68	NR	NR	NR	NR	NR	
**UAE**	500 W; 30 min	~0.69	NR	NR	18.42	NR	NR	
**DES**	Choline chloride:ethylene glycol (1:3)	~0.78	12.58	NR	8.00	NR	NR	
**STE**	Ethanol; 30 min	NR	NR	NR	NR	2.85 ± 0.08	18.57	[10]
**PEF**	Ethanol; 0.6 kV/cm	NR	NR	NR	NR	2.92 ± 0.11	11.94	
**UPAE**	Ethanol; 48 pulses/min; 60 W	NR	NR	NR	NR	3.11 ± 0.07	15.68	
**UBAE**	Ethanol; 37 kHz; two cycles	NR	NR	NR	NR	3.04 ± 0.06	17.34	
**SD**	Plant-to-water ratio 1:5 (*w*/*v*)	0.73	15.24	23.46	2.69	NR	NR	[104]
**MAHD**	450 W; 50 min	1.40	20.27	18.32	2.16	NR	NR	
**HD**	Water; 180 min	0.90	16.92	25.67	2.37	NR	NR	
**UAE**	20 kHz; 10 min	25.47	NR	NR	NR	53.91 ± 3.85	~120	[105]
**MAE**	950 W; 15 min	24.04	NR	NR	NR	58.93 ± 1.65	~132	
**SE**	70% ethanol; 240 min	23.18	NR	NR	NR	51.40 ± 0.50	~132	

Acronyms: **CHD**, Clevenger hydrodistillation; **HD**, hydrodistillation; **SD**, steam distillation; **MAHD**, microwave-assisted hydrodistillation; **DES-HD**, deep eutectic solvent-assisted hydrodistillation; **EAE**, enzyme-assisted extraction; **UAE**, ultrasound-assisted extraction; **UPAE**, ultrasound probe-assisted extraction; **UBAE**, ultrasound bath-assisted extraction; **STE**, stirring extraction; **PEF**, pulsed electric field-assisted extraction; **SE**, Soxhlet extraction; **TPC**, total phenolic content; **GAE**, gallic acid equivalents; **DW**, dry weight; **NR**, not reported. Direct comparisons should be made only among extraction methods evaluated within the same study. Values reported in different references should be interpreted independently because their experimental and analytical conditions were not standardized.

## Data Availability

No new data were created or analyzed in this study. Data sharing is not applicable to this article.

## Abbreviations

The following abbreviations are used in this manuscript:

AI	Artificial Intelligence
CHD	Clevenger Hydrodistillation
CO_2_	Carbon Dioxide
COSMO-RS	Conductor-like Screening Model for Real Solvents
DES	Deep Eutectic Solvent
DW	Dry Weight
EAE	Enzyme-Assisted Extraction
GAE	Gallic Acid Equivalent
HD	Hydrodistillation
HBA	Hydrogen Bond Acceptor
HBD	Hydrogen Bond Donor
MAE	Microwave-Assisted Extraction
MAHD	Microwave-Assisted Hydrodistillation
PEF	Pulsed Electric Field
QE	Quercetin Equivalent
RSM	Response Surface Methodology
SD	Steam Distillation
SE	Soxhlet Extraction
SFE	Supercritical Fluid Extraction
SFC	Supercritical Fluid Chromatography
TFC	Total Flavonoid Content
TPC	Total Phenolic Content
UAE	Ultrasound-Assisted Extraction
UBAE	Ultrasound Bath-Assisted Extraction
UPAE	Ultrasound Probe-Assisted Extraction
